# Comparison of Subcuticular Suture Materials in Cesarean Skin Closure

**DOI:** 10.1155/2015/141203

**Published:** 2015-08-27

**Authors:** Pınar Solmaz Hasdemir, Tevfik Guvenal, Hasan Tayfun Ozcakir, Faik Mumtaz Koyuncu, Gonul Dinc Horasan, Mustafa Erkan, Semra Oruc Koltan

**Affiliations:** ^1^Department of Obstetrics and Gynecology, Celal Bayar University Medical School, 45000 Manisa, Turkey; ^2^Department of Statistics, Celal Bayar University Medical School, Manisa, Turkey

## Abstract

*Aim.* Comparison of the rate of wound complications, pain, and patient satisfaction based on used subcuticular suture material.* Methods.* A total of 250 consecutive women undergoing primary and repeat cesarean section with low transverse incision were prospectively included. The primary outcome was wound complication rate including infection, dehiscence, hematoma, and hypertrophic scar formation within a 6-week period after operation. Secondary outcomes were skin closure time, the need for use of additional analgesic agent, pain score on numeric rating scale, cosmetic score, and patient scar satisfaction scale.* Results.* Absorbable polyglactin was used in 108 patients and nonabsorbable polypropylene was used in 142 patients. Wound complication rates were similar in primary and repeat cesarean groups based on the type of suture material. Skin closure time is longer in nonabsorbable suture material group in both primary and repeat cesarean groups. There was no difference between groups in terms of postoperative pain, need for additional analgesic use, late phase pain, and itching at the scar. Although the cosmetic results tended to be better in the nonabsorbable group in primary surgery patients, there was no significant difference in the visual satisfaction of the patients.* Conclusions.* Absorbable and nonabsorbable suture materials are comparable in cesarean section operation skin closure.

## 1. Introduction

Cesarean sections are one of the most commonly performed abdominal operations in women worldwide [1]. Wound healing is an important factor for lower complication rate and patient satisfaction in patients undergoing cesarean section.

Tully et al. showed that 73.9% of the obstetricians preferred to close skin with subcuticular sutures using Prolene (41.1%), Vicryl (17.5%) followed by dexon (13.5%), and staples (10.4%) [2]. The subcuticular absorbable sutures and surgical staples in cesarean wound closure were compared in the literature. Although there are conflicting results, closure with subcuticular suture materials were reported to be more advantageous in terms of wound healing, better cosmetic results and more patient satisfaction rates [3, 4].

The outcome of wound healing and patient satisfaction based on the use of subcuticular suture material (absorbable versus nonabsorbable) is unknown. The aim of this study is to compare the rate of wound complications, pain, and patient satisfaction based on used subcuticular suture material.

## 2. Materials and Methods

A total of 250 consecutive patients with viable pregnancies greater than 24 gestational weeks undergoing scheduled or unscheduled first or repeat cesarean delivery with low transverse incision were prospectively included between July 2014 and January 2015 at Celal Bayar University Hospital, Manisa, Turkey. The randomization of the patients to the groups was made by weekly alternating the type of suture (absorbable or nonabsorbable) used in cesarean operations. Obstetricians performing the operation were blind for the procedure characteristics including type of suture material, time needed for skin closure, and length of the wound. An inquiry form was filled by a resident from the study team the day after the operation and at the 6th weeks of follow-up. Wound infection was defined as any discharge, mild to severe requiring dressing and antibiotic use. Wound dehiscence was defined as separation of skin edges more than 1 cm in length. Hematoma was defined as wound swelling more than 1 cm in diameter accompanied by changing in colour of the skin. Hypertrophic scar was defined as pink-red coloured, hard, itchy, visible, and raised from the normal tissue level scar. Body mass index (BMI) was calculated at the time of delivery.

### 2.1. Exclusion Criteria

Patients with inability to obtain informed consent (emergent cases in which there was no time to get informed consent and patients who did not prefer to be in such a study protocol), fetal death, history of nonobstetric abdominal operation, known diabetes or gestational diabetes (except from abnormal glucose tolerance test values under control with diet only), any known immunological disorder, history of allergy for antibiotics and analgesics, and steroid drug usage were excluded. Patients implemented a nonroutine procedure (midline skin incision, postpartum hysterectomy or relaparotomy) because of an unexpected complication and patients who did not come for a second visit were also excluded.

The total number of the cesarean operations during the study period was 453 in our hospital. The main reasons for exclusion were lack of follow-up in 91 (20%), inability to obtain informed consent in 52 (11.8%), and presence of diabetes in 16 (3.5%) patients.

### 2.2. Ethical Consent

The study was approved by the Institutional Review Board of the Celal Bayar University with the number of 20.478.486-137, on March 26, 2014. Informed patient consent was obtained from the cases.

### 2.3. Operative Technique

Skin of the patients was cleaned with povidone iodine 3 to 4 minutes before the operation started. Prophylactic antibiotic (2nd generation cephalosporin) was administered in all patients right after cord clamping. The same operation technique (Pfannenstiel technique) was used for all patients. Subcutaneous tissues were closed with interrupted sutures (3.0 Vicryl Rapide [polyglactin 910]) in case of more than 1 cm subcutaneous tissue thickness. Polyglactin-910 (3.0 Vicryl) was used as absorbable and polypropylene (3.0 Prolen) was used as nonabsorbable suture material for skin closure. Continuous suturing with curved needle was used in all patients regardless of the suture type. Closure of the skin was performed by the attending physician who performed the operation and did not have information about the study protocol. Nonabsorbable suture materials were removed at postoperative 7 to 10 days. All patients included in the study were advised not to use any medication that would potentially affect wound healing.

Wound evaluations were initially performed at hospital discharge at postoperative day 2 or 3 and at 6th week of follow-up. The primary outcomes were complications related to wound healing (infection, dehiscence, hematoma, and hypertrophic scar formation) at 6th week of follow-up. Secondary outcomes were operative time, pain score on numeric rating scale (NRS) (0 = no pain; 2 = mild; 5 = moderate; 7 = severe; 10 = excruciating), itching at the scar site, cosmetic score (no scar or just a line, mild ridge with minimal change in colour, and presence of severe scar [>0,5 cm ridge and red in colour]), and patient scar assessment scale (1 = minimum and 10 = maximum). NRS, cosmetic score, and patient scar assessment scale were evaluated by asking the patient verbally to grade the extent and severity of the scar or pain on a scale of zero to ten for NRS and one to ten for patient scar assessment scale [5–8].

### 2.4. Statistical Analysis

Statistical analysis was performed with IBM SPSS Statistics 15.0 (SPSS Inc., Chicago, IL). A stratified analysis was made for patients with primary and repeat cesarean delivery. The Shapiro-Wilk test was used to calculate whether the numeric variables were normally distributed. For normally distributed variables, differences in the distributions of the patient characteristics were analyzed with Student's *t* test. The Mann-Whitney *U* test was used for abnormally distributed variables. Cross-tables and chi square analysis were employed in the evaluation of the categorical data. *P* value <0.05 was considered statistically significant.

## 3. Results

A total of 250 patients underwent cesarean section. Absorbable polyglactin-910 (3.0 Vicryl) was used in 108 (43.2%) and nonabsorbable polypropylene (3.0 Prolen) was used in 142 (56.8%) patients. Of the 250 patients, 167 underwent primary and 83 underwent repeat cesarean deliveries. Baseline characteristic including age, BMI, type, and length of skin incisions was similar in absorbable and nonabsorbable suture material groups for both primary and repeat cesarean patients (Table 1). Wound complication rates were similar in primary and repeat cesarean groups based on the type of suture material (Table 2). Skin closure time was longer in nonabsorbable suture material group in both primary and repeat cesarean patients (*P* = 0.016 and *P* = 0.035, resp.). There was no statistical difference between absorbable and nonabsorbable suture groups in terms of postoperative pain, need for additional analgesic use, itching, and pain at the scar tissue at 6th weeks follow-up (Table 3). Although the cosmetic results tended to be better in nonabsorbable group in primary surgery patients (*P* = 0.089), there were no significant differences in the visual satisfaction of the patients (*P* = 0.717) (Table 3).

## 4. Discussion

Functional and cosmetic aspects of cesarean surgeries gain increasing importance in recent years. There is still a lack of data in terms of the best method for skin closure in cesarean operations [9, 10]. There are several studies in the literature comparing staples with suture in closure of cesarean incisions [3, 7, 10–13]. A meta-analysis which included 877 women from 5 studies compared the use of staples and subcuticular sutures. Study results showed that wound dehiscence and complication rates increased with staples, although the operation time was shortened only by a mean of 5.05 minutes. The authors recommended that subcuticular closure of the skin should be preferred [12]. Similar results were found by Mackeen et al. in 2015 [13]. Frishman et al. compared the staples with absorbable subcuticular suture in 66 women undergoing cesarean section and reported that operation time was significantly shorter with the use of staples. But the use of absorbable subcuticular suture resulted in less pain and use of lower dose of analgesics [11, 14]. A 2012 Cochrane review reported that staples and subcuticular absorbable sutures were similar in terms of wound infection and wound complication rates except that the incidence of wound dehiscence was increased with early (<4 days) removal of staples in women with Pfannenstiel incisions [9].

According to a recent prospective, randomized study closing cesarean incisions with suture is associated with 57% decrease in wound complications compared to closure with staples [13] along with better patient satisfaction rates [15]. Gaertner et al. compared subcuticular sutures with staples in both subcuticular layer closure and nonclosure group of patients and found no significant difference among the groups in terms of wound complications and patient satisfaction at 4th month of follow-up [16].

Based on the results of the abovementioned studies, subcuticular sutures seem to be more advantageous compared to staples [3, 12, 13]. But there is a lack of data comparing the outcome of different types of subcuticular suture materials. Tan et al. conducted a study comparing the suture materials and reported that absorbable and nonabsorbable sutures have similar short-term outcomes but nonabsorbable sutures have a disadvantage of requirement of removal. Additionally, late-term itching at the scar site was seen more frequently in absorbable suture material group possibly due to the late absorption of this kind of suture material [17]. This study was a randomized, controlled study comparing absorbable (poliglecaprone 25) and nonabsorbable (polypropylene) suture materials in low-transverse incisions. Inclusion of obstetric and nonobstetric cases as well as diabetic cases was the downside of this study. In our study, we compared the most commonly used suture materials (Vicryl and Prolen) [2] just in cesarean sections and performed a stratified analysis for first and repeat cesarean patients. In addition, we excluded patients with diabetes which is an important confounder in wound healing.

### 4.1. Study Limitations

The major limitation of this study was the difference in the number of the patient population in the study groups despite the fact that we expected them to be similar when making the sample size calculations. However this was due to the weekly randomization process and was not expected to have confounder effect on the results of our study because patient characteristics such as age, BMI, and wound length were found similar.

## 5. Conclusion

Our results showed that there was no significant difference in terms of wound complications. There is a tendency to get better wound healing with nonabsorbable suture materials, although this difference did not affect the patient's satisfaction rate.

## Figures and Tables

**Table 1:** Descriptive statistics of study groups.

	Primary Cesarean			Repeat Cesarean		
	Absorbable (*n* = 80)	Nonabsorbable (*n* = 87)	*P* value	Absorbable (*n* = 28)	Nonabsorbable (*n* = 55)	*P* value
Age (years) (mean ± SD)	26.78 ± 4.83	27.11 ± 5.35	0.678^*∗*^	30.71 ± 5.36	29.16 ± 4.92	0.192^*∗*^
BMI (mean ± SD)	27.24 ± 4.48	28.38 ± 4.88	0.130^*∗*^	30.12 ± 5.25	29.29 ± 4.92	0.487^*∗*^
Wound length (mm)						
mean ± SD	11.00 ± 1.36	11.27 ± 1.70		11.03 ± 1.29	11.20 ± 1.67	
Median (25th–75th)	11 (10–12)	11 (10–12)	0.813^*∗∗*^	11 (10–12)	11 (10–12)	0.775^*∗∗*^

^*∗*^Student's *t* test, ^*∗∗*^Mann-Whitney *U* test.

**Table 2:** Comparison of the groups in terms of the primary outcomes (complication rates).

Complication	Primary Cesarean			Repeat Cesarean		
	Absorbable (*n* = 80)	Nonabsorbable (*n* = 87)	*P* value	Absorbable (*n* = 28)	Nonabsorbable (*n* = 55)	*P* value
Wound infection (%)	22.5%	14.9%	0.210^*∗*^	14.3%	12.7%	1.000^*∗∗*^
Hematoma	6.3%	3.4%	0.480^*∗∗*^	0.0	5.5%	0.546^*∗∗*^
Dehiscence	5.0	5.7	1.000^*∗∗*^	10.7	10.9	1.000^*∗∗*^
Hypertrophic scar	3.8%	2.3%	0.668^*∗∗*^	3.6%	0.0	0.352^*∗∗*^

^*∗*^Chi square test, ^*∗∗*^Fisher's exact test.

**Table 3:** Comparison of the groups in terms of the secondary outcomes.

	Primary Cesarean			Repeat Cesarean		
	Absorbable (*n* = 80)	Nonabsorbable (*n* = 87)	*P* value	Absorbable (*n* = 28)	Nonabsorbable (*n* = 55)	*P* value
Skin closure time						
Mean ± SD	6.77 ± 1.12	7.31 ± 1.23		7.3 ± 0.97	7.83 ± 1.04	
Median (25th–75th)	7 (6–8)	7 (7-8)	0.016^*∗∗*^	7 (7-8)	8 (7–9)	0.035^*∗∗*^
Analgesic use	12.5%	30.2%	0.006^*∗*^	21.4%	29.1%	0.455^*∗*^
Postoperative pain (VAS)						
Mean ± SD	3.40 ± 2.74	4.02 ± 2.66		4.0 ± 3.30	4.11 ± 2.69	
Median (25th–75th)	3 (1–5)	3 (2–6)	0.099^*∗∗*^	4 (1–6.75)	4 (2–6)	0.652^*∗∗*^
Pain at 6th week	11.3%	21.8%	0.067^*∗*^	28.6%	27.3%	0.901^*∗*^
Itching	3.8%	3.4%	1.000^*∗∗∗*^	0.00	0.00	NA
Cosmetic results			0.089^*∗*^			0.723^*∗*^
No scar or just a line	41.3%	56.3%		57.1%	63.6%	
Mild ridge with minimal change in colour	52.5%	35.6%		35.7%	27.3%	
Severe scar (>0,5 cm ridge and red in colour)	6.3%	8.0%		7.1%	9.1%	
Satisfaction						
Mean ± SD	6.70 ± 2.62	6.56 ± 2.61		7.28 ± 2.44	7.05 ± 2.79	
Median (25th–75th)	8 (5–9)	7 (5–9)	0.717^*∗∗*^	8 (5.25–9.75)	8 (5–10)	0.879^*∗∗*^

^*∗*^Chi square, ^*∗∗*^Mann-Whitney *U* test, ^*∗∗∗*^Fisher's exact test, and NA: not applicable.
